# Expression of phosphorylated extracellular signal-regulated kinase at the invasive front of hepatic colorectal metastasis

**DOI:** 10.3892/ol.2015.2874

**Published:** 2015-01-14

**Authors:** HYUN-SOO KIM, SUNG-IM DO, BYEONG-JOO NOH, YOUNG IN JEONG, SUN JIN PARK, YOUN WHA KIM

**Affiliations:** 1 Department of Pathology, Yonsei University College of Medicine, Seoul 120-752, Republic of Korea; 2 Department of Aerospace Medicine, Republic of Korea Air Force Aerospace Medical Center, Cheongju 363-849, Republic of Korea; 3 Department of Pathology, Kangbuk Samsung Hospital, Sungkyunkwan University School of Medicine, Seoul 110-746, Republic of Korea; 4 Department of Pathology, Kyung Hee University Graduate School of Medicine, Seoul 130-701, Republic of Korea; 5 Department of Surgery, Kyung Hee University Graduate School of Medicine, Seoul 130-701, Republic of Korea

**Keywords:** extracellular signal-regulated kinase, invasive tumor front, Raf kinase inhibitor protein, colorectal carcinoma, liver, metastasis

## Abstract

Raf-1 kinase inhibitory protein (RKIP), an endogenous inhibitor of the extracellular signal-regulated kinase (ERK) pathway, suppresses metastasis in a number of cancer types, including colorectal carcinoma (CRC); thus, RKIP downregulation significantly contributes to CRC invasiveness and metastatic potential. However, our previous study demonstrated that RKIP-positive tumors in CRC patients are predictive of hepatic colorectal metastases (HCMs). Based on the previous finding that the ERK pathway can be activated independently of RKIP, we hypothesized that RKIP-expressing HCMs may express significant levels of phosphorylated ERK (pERK). Thus, the present study evaluated the expression of RKIP and pERK in 68 HCM tissue samples using immunohistochemistry. RKIP expression was positive in 22 (32.4%) of the 68 samples, seven (31.8%) of which exhibited nuclear pERK immunoreactivity exclusively at the invasive tumor front. Furthermore, pERK expression at the invasive front was significantly associated with recurrent HCM following hepatic resection, and pERK expression observed at the invasive front of RKIP-expressing HCMs indicated that the activation of the ERK pathway may also be involved in the invasive process of these tumors, despite the presence of RKIP. A strong association between pERK expression and the presence of recurrent HCM may indicate that the ERK pathway is important in the metastatic recurrence of RKIP-positive HCM.

## Introduction

Colorectal carcinoma (CRC) is one of the most common types of solid malignancy and a main cause of cancer-related mortality globally. Colorectal carcinogenesis is a complex multistep process that involves progressively disrupting intestinal epithelial cell proliferation, differentiation, apoptosis and survival mechanisms (1,2). Mitogen-activated protein kinases (MAPKs), members of a large family of serine-threonine kinases, form major cell proliferation signaling pathways from the cell surface to the nucleus (3), and can be activated in response to a wide range of extracellular stimuli (4). The first member of this family to be characterized was extracellular signal-regulated kinase (ERK). The ERK pathway is important for cell survival and proliferation, and a number of key growth factors and proto-oncogenes act via this cascade, transducing the signals to promote cell growth and differentiation (5). For example, activated Ras activates Raf-1, which in turn phosphorylates and activates MAPK/ERK kinase (MEK), resulting in activation of ERK, the only known substrate of MEK. Analysis of established cell lines indicates that constitutive activation of MAPK is induced in tumors in a tissue-specific manner; for example, tumor cells derived from CRC tissues exhibit a particularly high frequency of ERK activation (6). Furthermore, increasing evidence indicates that the ERK pathway is important for regulating apoptosis and cell proliferation, and that its activation is involved in the tumorigenesis, progression and oncogenic behavior of CRC (7–12).

Raf-1 kinase inhibitory protein (RKIP), a member of the phosphatidylethanolamine binding protein family, was initially identified as an endogenous inhibitor of the ERK pathway. By binding to Raf-1 or MEK, RKIP interferes with MEK activation by Raf-1, thus inhibiting ERK activation (13). Our previous studies demonstrated that RKIP downregulation significantly contributes to the invasiveness and metastatic potential of a number of different types of human carcinoma, including CRC (14–19); for example, it was identified that RKIP expression was significantly reduced in metastatic tumor tissues compared with corresponding primary tumor and non-neoplastic tissues (15,17,18,20). In addition, a reduction in RKIP expression levels was associated with lymphovascular invasion, as well as nodal and distant metastases, increased local recurrence, advanced tumor stage and decreased survival rate (14–16,18). Furthermore, RKIP downregulation appeared to correlate with aggressive oncogenic behavior. Thus, the data indicated that RKIP may act as a suppressor of metastasis in CRC.

However, our previous studies determined conflicting results regarding the association between RKIP expression and metastasis in specific CRC patients, for example, hepatic colorectal metastasis (HCM) was histopathologically diagnosed in CRC patients whose tumors exhibited positive RKIP immunoreactivity (19). This finding indicated that metastatic properties are preserved in a certain group of tumor cells despite the presence of the metastatic suppressor RKIP. Furthermore, previous studies demonstrated that ERK can be phosphorylated regardless of RKIP status (21,22). Based on these data, we hypothesized that the ERK pathway may be activated in RKIP-expressing HCM tissues, and that the activation of this pathway may be involved in the invasive process of HCM cells.

Therefore, the present study investigated whether phosphorylated ERK (pERK) is detectable in RKIP-expressing HCM tissues. In addition, the association between pERK expression and various clinicopathological characteristics of HCM patients was evaluated to assess its clinical value.

## Patients and methods

### Patient and tissue specimens

Human HCM tissue samples were obtained from 68 consecutive patients (46 male and 22 female; median age, 61 years; mean age, 60.1 years) who underwent surgery at the Kyung Hee University Hospital (Seoul, Korea) between January 2005 and December 2012. All of the patients met the following criteria for hepatic resection with intent to cure: i) No signs of extrahepatic metastases in pre-operative imaging, including chest X-ray, abdominal ultrasonography and abdominopelvic computed tomography; ii) HCM that allowed adequately sized, well-vascularized hepatic remnants to remain following hepatic resection; and iii) medical fitness for major hepatic resection. Only patients whose metastases were resectable on presentation were included in the present study. The resected lesions were stained with hematoxylin and eosin and reviewed by two independent pathologists, who selected the most representative slide from each case for subsequent immunohistochemical staining. The selected tissues were fixed in 10% neutral buffered formalin for 24–72 h at room temperature for paraffin embedding prior to analysis. Furthermore, the following clinicopathological data was assessed in each case: Patient age and gender, size, number and distribution of HCMs, HCM recurrence and survival data obtained from follow-up after hepatic resection. Informed consent was obtained from all participants. The study was approved by the institutional review board of the Republic of Korea Air Force Aerospace Medical Center (Cheongju, Korea).

### Immunohistochemistry

RKIP and pERK protein expression was assessed by immunohistochemistry using the Bond Polymer Intense Detection System (Leica Biosystems, Seoul, Korea), according to the manufacturer’s instructions. Briefly, 4-μm sections of formalin-fixed, paraffin-embedded tissue were deparaffinized with Bond Dewax solution (Leica Biosystems) and an antigen retrieval procedure was performed using Bond Epitope Retrieval solution 1 (cat. no. AR9961; Leica Biosystems) for 30 min at 100°C. Endogenous peroxidases were blocked by incubation with hydrogen peroxide for 5 min and the sections were incubated for 15 min at ambient temperature with a 1:200 dilution of rabbit polyclonal anti-RKIP (cat. no. sc-32623; Santa Cruz Biotechnology, Inc., Santa Cruz, CA, USA) or rabbit monoclonal anti-pERK (phospho-p44/42 MAPK [Thr202/Tyr204; clone, 20G11]; cat. no. 4376S; Cell Signaling Technology, Inc., Beverly, MA, USA). Staining was performed using a biotin-free polymeric horseradish peroxidase-linker antibody conjugate system with the Bond Max automatic slide stainer (Leica Biosystems), and the slides were visualized with 3,3′-diaminobenzidine (DAB) solution [1 mm DAB, 50 mm Tris-HCl buffer (pH 7.6) and 0.006% H_2_O_2_]. In addition, the nuclei were counterstained with hematoxylin and the slides were dehydrated through a series of graded alcohols (70, 90 and 100%), cleared in xylene (Sigma Aldrich, St. Louis, MO, USA) and sealed with coverslips. Positive control samples consisted of healthy colonic mucosa for RKIP, and prostate carcinoma and malignant melanoma for pERK (23,24). The negative control was prepared by substituting non-immune serum for primary antibody and resulted in no detectable staining.

### Evaluation of immunohistochemical staining

Two experienced independent pathologists, who were blinded to the clinicopathological data and outcomes of the patients, examined the immunohistochemical expression of RKIP and pERK in the stained sections. Immunoreactivity for RKIP and pERK was analyzed using previously described semi-quantitative scoring methods (14–20), and the scores of the two pathologists were compared, with discrepancies resolved by re-examination by the two pathologists to achieve a consensus score. The final score was the sum of the scores for the percentage of positive tumor cells (0, 0%; 1, ≤25%; 2, 25–50%; and 3, >50%) and the staining intensity (0, absent; 1, faint; 2, moderate; 3, strong). Additionally, expression scoring was follows: 0–2, negative expression; 3–4, weak expression; and 5–6, positive expression. All the immunostaining scores were determined at the center, intermediate zone and invasive front of the tumor, as well as at subcellular locations.

### Statistical analysis

Fisher’s exact test was performed to determine whether pERK expression was associated with each HCM clinicopathological characteristic, and a survival analysis was used to examine the prognostic significance of pERK expression in the HCM patients by producing survival curves using the Kaplan-Meier method and applying a log-rank test to analyze any statistical differences. All statistical analyses were performed using SPSS software (version 15.0; SPSS Inc., Chicago, IL, USA) and P<0.05 was used to indicate a statistically significant difference.

## Results

**Figure 1:**
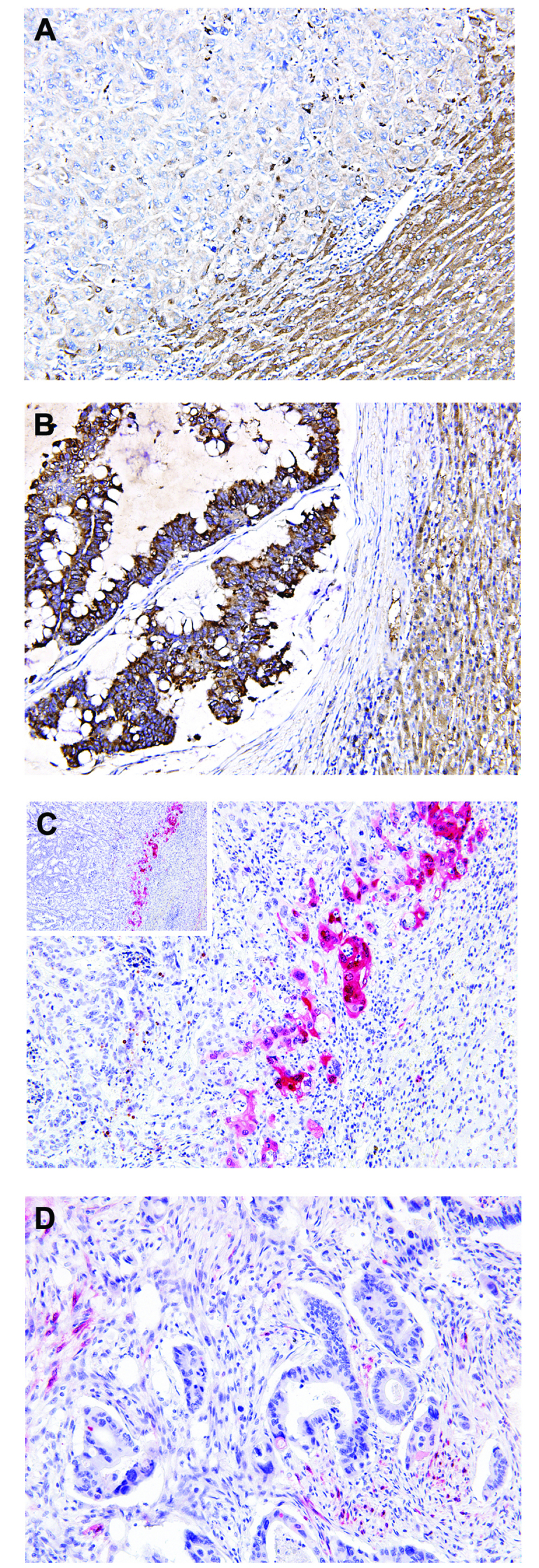
Immunoreactivity for Raf-1 kinase inhibitory protein (RKIP) and phosphorylated extracellular signal-regulated kinase (pERK) in hepatic colorectal metastasis (HCM) samples. (A) Negative RKIP expression in tumor cells; and (B) positive RKIP expression in the cytoplasm of tumor cells, with adjacent hepatocytes (right lower corner) used as the internal positive controls. (C) Positive pERK expression in tumor cells demonstrating selective expression of pERK at the invasive front of HCM, but not the inner areas. pERK expression formed a band-like pattern (inset) and localization of pERK was accompanied with loss of the epithelial phenotype, characterized by the detachment of small isolated clusters of tumor cells and dedifferentiation. Tumor cells invading the hepatic parenchyma exclusively expressed pERK in the nucleus and cytoplasm. (D) Negative pERK expression in tumor cells. (stain, polymer method; A–D, magnification, ×200).

Overall, RKIP immunoreactivity appeared to be predominantly cytoplasmic and homogeneous throughout the tumor, with protein expression levels determined to be negative in 36.8% (25/68; Fig. 1A), weak in 30.8% (21/68) and positive in 32.4% (22/68; Fig. 1B) of samples. Of the 22 RKIP-expressing cases, seven (31.8%) exhibited intense nuclear and moderate cytoplasmic pERK immunoreactivity (Fig. 1C), while the remaining 15 cases (68.2%) exhibited no pERK immunoreactivity (Fig. 1D). In contrast to RKIP expression, pERK immunoreactivity was heterogeneous and strongly positive pERK protein expression was exclusively detected at the invasive tumor front, whereas the tumor center and intermediate zones did not display pERK immunoreactivity. Furthermore, only cells that faced the hepatic parenchyma expressed pERK, forming a band-like pattern (Fig. 1C). This unique localization of pERK was accompanied by loss of the epithelial phenotype, and characterized by the detachment of small isolated clusters of tumor cells and a dedifferentiated morphology at the invasive front. By contrast, cells in the center and intermediate zones of the tumor exhibited a typical epithelial growth pattern characterized by adherent polarized cells forming clear trabecular or tubular structures. In addition, pERK staining exhibited strong staining intensity in the nucleus, consistent with ERK activity being highest in this subcellular location.

The association between pERK expression at the invasive front and the clinicopathological characteristics of HCM is indicated in Table I. It was observed that pERK expression at the invasive front was significantly associated with recurrent HCM (P<0.001); all patients with pERK-positive tumors developed recurrent HCM, while in patients who did not develop recurrent HCM, pERK expression was absent. The prediction of recurrent HCM was associated with pERK expression at the invasive front, with 87.5% sensitivity, 100% specificity, a 100% positive predictive value and a 93.3% negative predictive value.

## Discussion

Histopathological studies have demonstrated that invasiveness, characterized by the loss of epithelial differentiation and the acquisition of a mesenchymal phenotype, is predominantly observed at the periphery of CRC tumors (25–27), with cells at the invasive front considered to exhibit a higher malignant potential than the inner areas of the same tumor. Previous studies utilized CRC cell lines and surgical specimens to demonstrate that the ERK pathway is critical for the invasive and proliferative properties of these tumors (6,25,28,29). Furthermore, pERK was expressed at higher levels in more advanced stages of CRC, demonstrating the importance of this molecule in CRC progression, invasion and metastasis (2). In the present study, the selective localization of pERK was identified at the invasive front of a subpopulation of metastasized CRC cells, but not in the inner, more differentiated areas of the tumor, and pERK expression was accompanied by the loss of the epithelial phenotype. This pERK expression pattern is consistent with the results of the aforementioned studies and possibly reflects the high biological aggressiveness of tumor cells at the invasive edge. Activation of the ERK pathway initiates transcription, which leads to the expression of matrix metalloproteinase and myosin light-chain kinase, inducing degradation of the basement membrane and promoting cell migration and invasion (30–32). Additionally, recent studies into the functional role of pERK in carcinomas revealed that pERK upregulation augmented cell motility on extracellular matrix components, increased Matrigel invasion and promoted the growth of the tumor (33–35). To the best of our knowledge, the distinct distribution of pERK at the invasive front of HCM has yet to be reported, and the findings of the present study indicate that pERK is significant in the HCM invasive process *in vivo*, which supports the previous finding that activation of the ERK pathway is necessary for the induction of epithelial cell dedifferentiation (36).

Furthermore, the current study detected intense pERK immunoreactivity in RKIP-expressing metastatic tumors. Although oncogenic pathways other than the ERK cascade may contribute to CRC cell dissemination in RKIP-expressing tumors, the possibility that the ERK pathway may be involved in the metastatic process regardless of RKIP status must be considered, thus, the present study proposes a number of possible explanations for this result. Firstly, the ERK cascade is not a simple linear pathway, but has numerous positive and negative regulatory components, which function at all levels of the cascade (37). For example, a previous study demonstrated that RKIP regulates Raf-1, but not B-Raf, indicating that B-Raf is able to phosphorylate and activate MEK and ERK independent of RKIP (22). Papin *et al* (21) reported that B-Raf displayed higher MEK kinase activity compared with Raf-1. Based on these data, the present study proposes that *BRAF* mutations may be involved in ERK activation in RKIP-expressing tumors. Secondly, in addition to the predominant MEK activators Raf-1 and B-Raf, MEK kinase-1 and A-Raf are able to phosphorylate MEK and thus activate the ERK pathway, although the biochemical potency of A-Raf is much weaker than that of Raf-1 or B-Raf (38). Finally, MEK is capable of autophosphorylation, resulting in an increase in MEK kinase activity (39). Thus, although the observation of pERK expression at the invasive front of RKIP-expressing HCMs indicates that the activation of the ERK pathway contributes to the invasive process in RKIP-expressing metastatic tumor cells, additional studies are required to clarify or disprove the findings of the present study.

The treatment of primary CRC with surgical resection, combined with chemotherapy or radiation therapy in certain cases, is curative in a number of patients, however, almost half will develop HCM during the disease course (40). Furthermore, even when hepatic resection is performed with curative intent, the majority of patients experience tumor recurrence (41); one-third of which develop recurrent metastases isolated to the liver. Repeat hepatic resection for recurrent HCM appears to be warranted in carefully selected patients due to the reasonable survival expectations, similar to those of patients undergoing single hepatic resection (42). In this regard, identifying markers based on biological determinants may facilitate the improvement and earlier stratification of patients according to recurrence risk, and aid in selecting patients who may benefit from repeated hepatic resection. The present study demonstrated that pERK expression was significantly associated with recurrent HCM, and high sensitivity, specificity and predictive values indicated that pERK expression at the invasive front of HCM may be used as a novel biomarker for the prediction of recurrent HCM.

In conclusion, the present study identified the unique, heterogeneous expression of pERK in HCM and its association with metastatic recurrence. The selective upregulation of pERK at the invasive tumor front indicates that activation of the ERK pathway is involved in the invasive process of HCM cells. In addition, pERK expression at the invasive front of HCM was strongly associated with HCM recurrence, with high sensitivity, specificity and predictive values. These findings indicate that pERK expression may be used as a novel predictive biomarker of metastatic recurrence and an effective target for therapeutic strategies against RKIP-expressing HCM.

## Figures and Tables

**Table I:** Association between pERK expression at the invasive front and clinicopathological characteristics of HCM patients.

	pERK status		
		
Characteristic	Positive (n=7)	Negative (n=15)	P-value
Age, n (%)			
≥61 years	4 (57.1)	6 (40.0)	0.652
<61 years	3 (42.9)	9 (60.0)	
Gender, n (%)			
Male	5 (71.4)	10 (66.7)	1.000
Female	2 (28.6)	5 (33.3)	
HCM, n (%)			
Single	4 (57.1)	11 (73.3)	0.630
Multiple	3 (42.9)	4 (26.7)	
HCM size, n (%)			
≥2.5 cm	3 (42.9)	8 (53.3)	1.000
<2.5 cm	4 (57.1)	7 (46.7)	
HCM distribution, n (%)			
Unilobar	6 (85.7)	12 (80.0)	1.000
Bilobar	1 (14.3)	3 (20.0)	
HCM recurrence, n (%)			
Present	7 (100.0)	1 (6.7)	<0.001^a^
Absent	0 (0.0)	14 (93.3)	
Median survival (range), months^b^	26 (1–62)	28 (6–85)	0.853

a Statistically significant.

pERK, phosphorylated extracellular signal-regulated kinase; HCM, hepatic colorectal metastasis.
